# Knowledge, attitudes, and practices of Saudi physicians toward the use of SGLT-2 inhibitors and GLP-1 receptor agonists in patients with type 2 diabetes mellitus: a nationwide cross-sectional study

**DOI:** 10.3389/fendo.2026.1929125

**Published:** 2026-08-27

**Authors:** Marwan A. Alrasheed, Bader M. Alhadhrami, Abdulaziz A. Alqarni, Nawaf M. Alghamdi, Omar A. Alfurayh, Omar A. Almohammed

**Affiliations:** 1 Department of Clinical Pharmacy, College of Pharmacy, King Saud University, Riyadh, Saudi Arabia; 2 Pharmacoeconomics Research Unit College of Pharmacy, King Saud University, Riyadh, Saudi Arabia

**Keywords:** attitudes, clinical inertia, GLP-1 receptor agonists, knowledge, physicians, practices, SGLT-2 inhibitors, type 2 diabetes mellitus

## Abstract

**Background:**

Despite guidelines recommending sodium-glucose cotransporter-2 inhibitors (SGLT-2i) and glucagon-like peptide-1 receptor agonists (GLP-1RA) for type 2 diabetes mellitus (T2DM) patients with cardiovascular risk, utilization remains low. This study evaluated the knowledge, attitudes, and practices (KAP) of physicians in Saudi Arabia toward these agents.

**Methods:**

A nationwide cross-sectional study was conducted among 399 physicians in Saudi Arabia between December 2024 and November 2025. Participants completed an online survey assessing pharmacological knowledge, their attitude, and prescribing practices. Multivariable logistic regression was used to identify independent determinants of KAP outcomes.

**Results:**

Theoretical knowledge was high, with 91.0% of physicians demonstrating adequate knowledge of SGLT-2i and 78.4% for GLP-1RA. Conversely, only 23.6% held an overall positive attitude, while 75.4% met the threshold for appropriate practice. Notably, 90.0% of physicians identified medication availability and patient accessibility as constant prescribing barriers. Multivariable analysis showed that while knowledge scores were generally high, they did not independently guarantee a positive attitude. Older age was significantly associated with higher odds of inappropriate practice, whereas specialist status and higher attitude scores were linked to better clinical practices.

**Conclusion:**

The study highlights a significant dissociation between high theoretical knowledge and attitudinal hesitancy among Saudi physicians. These findings suggest that under-prescribing may be associated more with attitudinal and structural factors, such as limited medication access, than with knowledge deficits. Future interventions should prioritize building clinical confidence through academic detailing and addressing systemic access issues to optimize evidence-based management for patients with T2DM.

## Introduction

Managing type 2 diabetes mellitus (T2DM) is challenging due to the need for continuous therapeutic review and monitoring, particularly among patients with comorbid atherosclerotic cardiovascular disease (ASCVD) (1). Over the years, management has shifted toward incorporating agents with cardioprotective and nephroprotective effects. In 2018, the European Association for the Study of Diabetes (EASD) in collaboration with the American Diabetes Association (ADA) jointly recommended sodium-glucose cotransporter-2 inhibitors (SGLT-2i) and glucagon-like peptide-1 receptor agonist (GLP-1RA) as preferred classes of medications for glycemic control in patients with uncontrolled T2DM and ASCVD (2). This recommendation was based mainly on data from several large-scale cardiovascular outcome trials (CVOTs), including CANVAS, EMPA-REG OUTCOME, LEADER, SUSTAIN-6 and others, which demonstrated the cardioprotective benefits of multiple medications from two groups, SGLT-2i and GLP-1RA (3–6). These medications not only provided favorable cardiovascular outcomes but also reduced hyperglycemia and body weight while lowering the incidence of hypoglycemia (7).

Despite strong clinical evidence, uptake of these agents remains below guideline expectations, and prescribing has historically lagged in eligible patients across multiple health systems (1). Early real-world data illustrate the magnitude of this gap: in a United States cohort spanning 2013 to 2016, only 7.2% of pharmacologically treated patients with T2DM received an SGLT-2i, and, paradoxically, those with myocardial infarction, heart failure, or kidney disease were less likely to be treated (8). Because these estimates predate the marked expansion in use that followed the cardiovascular and renal outcome trials, they should be interpreted as historical baselines rather than current utilization. More recent analyses confirm that, although prescribing has risen substantially, it remains well short of guideline targets. In United States ambulatory cardiovascular care, SGLT-2i use for heart failure increased from 4.6% in 2019 to 16.2% in 2023 (9), and among adults with type 2 diabetes and atherosclerotic cardiovascular disease, combined SGLT-2i and GLP-1RA use remained modest through 2024 (10). A multi-specialty analysis similarly found that only 17.4% of eligible patients were prescribed either class (11). Similarly, a study conducted in the United Kingdom indicated that although the use of SGLT-2i medications was on the rise, their overall utilization remains low, and prescriptions were not necessarily based on these patients’ cardiovascular status (12). Another study in the United States highlighted the very low utilization of SGLT-2i among patients with T2DM and CKD (13).

In Hong Kong, despite the government subsidies for SGLT-2i prescriptions, the adoption rate for these medications is still lower than expected, suggesting some other barriers to utilization (14). In Saudi Arabia, a multicenter retrospective cross-sectional study noted a similar trend and reported that only 19% of patients with T2DM who were eligible for SGLT-2i or GLP-1RA therapy were prescribed these agents (15). More recently, a tertiary-center analysis of older adults with T2DM in Saudi Arabia found that only 7.4% of the overall outpatient cohort received an SGLT-2i or GLP-1RA, and only 1.6% were identified as having a qualifying cardiovascular or renal indication, underscoring persistent under-recognition and under-treatment (16).

Physician decisions regarding SGLT-2i and GLP-1RA use may be influenced by several factors, and unique barriers exist across specialties such as endocrinology, primary care, and cardiology (17). Additionally, knowledge gaps regarding CVOTs may contribute to lower prescribing rates among physicians, potentially impacting the broader adoption of these therapies (18, 19). Limited knowledge about these medications was recognized as a significant barrier to the optimal utilization of SGLT-2i in clinical settings (17, 20). Similarly, a small study conducted in a southern region of Saudi Arabia reported a low level of knowledge among physicians as a major factor contributing to clinical inertia (21). Thus, the study suggested that further research with a larger sample size, broader scope, and focus on knowledge, attitudes, and actual prescribing practices is necessary. This would provide valuable insights that could lead to improvements in diabetes management and patient care.

Therefore, this study aims to evaluate physicians’ understanding of the pharmacological mechanisms, benefits, and risks of SGLT-2i and GLP-1RA, explore their perceptions and attitudes towards these medications, and assess their current prescribing practices of these medications. Additionally, it seeks to identify gaps in knowledge and practices to improve the effective and evidence-based prescribing for these medications in clinical practice.

## Methods

### Study design and participants

A cross-sectional questionnaire-based study was conducted to assess the knowledge, attitude, and practice (KAP) toward the use of SGLT-2i and GLP-1RA for patients with T2DM among physicians providing care to these patients in Saudi Arabia. Physicians from multiple public and private hospitals providing care to patients with T2DM in Saudi Arabia were invited to participate in the study. The study included physicians actively practicing and regularly managing patients with T2DM at the time of the study. These physicians primarily specialized in or worked within primary care, family medicine, internal medicine, endocrinology, nephrology or cardiology clinics. Participants were excluded if they were not providing care to patients with T2DM. The participants’ KAP toward the use of SGLT-2i and GLP-1RA was assessed using a comprehensive online questionnaire that was developed for the purpose of the study. The study protocol was approved by the Institutional Review Board (IRB) at King Saud University Medical City (Ref. No. 24/1562/IRB).

### Questionnaire

The questionnaire consisted of four parts (Appendix A). The first part included questions about the demographic and professional characteristics of the participants, covering age, gender, professional status, specialty, and years of experience, and region of practice. Age was assessed as a categorical variable, dividing participants into five groups: 25-35, 36-45, 46-55, 56-65, and over 65 years. Gender (male vs. female) and nationality (Saudi vs. non-Saudi) were collected as dichotomous variables. Professional status was categorized as general practitioner, resident, specialist, or consultant. Specialty information included family medicine, internal medicine, endocrinology, cardiology, and nephrology. Years of experience were grouped into four categories: less than 5, 5-10, 11-15, and over 15 years. While region of practice setting was classified as Riyadh and others.

The other three parts in the questionnaire included questions about the KAP of the participants toward the use of SGLT-2i and GLP-1RA. The knowledge section aimed to evaluate the participants’ understanding of the basic pharmacological information, as well as the clinical benefits and risks associated with SGLT-2i and GLP-1RA use in patients with T2DM. The attitude section assessed perceived utilization, concerns, impact on morbidity and mortality, prescribing hesitancy, and confidence in discussing benefits and risks. It also examined medication preferences and their influence on patient adherence and quality of life. The questions assessed their consideration and adherence to protocols. They also explored factors influencing prescriptions, such as risk, weight loss needs, patient preferences for administration, medication availability, as well as the frequency of initial therapy use and barriers to prescribing.

The questionnaire was developed by the research team after reviewing the literature about the topic especially the American Diabetes Association guideline (22). Two junior authors (BA and AA) drafted the questionnaire, and two senior authors (OA and MA) revised it individually. Then, the four authors revised the items on the questionnaire one-by-one for clarity and validity as a group in multiple meetings till consensus was reached for all items. The questionnaire was then revised by seven independent physicians who usually provide care to patients with T2DM to ensure the face validity of the scale and the appropriateness of the items for the targeted physicians. The authors in the study were two junior pharmacists, and the two senior authors were academicians in a college of pharmacy. Overall, 11 healthcare providers have individually or collaboratively revised the items in multiple rounds to ensure the validity of the questionnaire.

The questionnaire was then sent to a small group of physicians (n = 20) to pilot-test the questionnaire and ensure its clarity for the targeted audience and their feedback was positive about the items and only minor changes were made at that time and no item was removed from the questionnaire at that stage. The final KAP sections on the questionnaire had acceptable internal reliability for most scales: KR-20 = 0.61 for SGLT-2i knowledge, KR-20 = 0.37 for GLP-1RA knowledge, Cronbach’s α = 0.70 for attitude, and Cronbach’s α = 0.80 for practice.

### Assessment of KAP of the participants

For the knowledge construct, eight items for each class of medication were asked and participants received 1 point for each correct answer and 0 for incorrect/not sure answers. Participants with at least 5 out of 8 points (>60%) for each medication group (SGLT-2i and GLP-1RA) were considered to have adequate knowledge about the use of that group in patients with T2DM. For the attitude construct, nine items were asked to assess the participants’ attitude and beliefs about medications in either one of these two groups (SGLT-2i and GLP-1RA). Participants received points based on their responses: +2 points for “Totally Agree,” +1 point for “Agree,” 0 point for “Neutral,” -1 point for “Disagree,” and -2 points for “Totally Disagree.” Participants with a total score of at least 11 points (>60%) were considered to have an overall positive attitude about these medications. For the practice construct, ten items were asked to assess the participants’ safe and appropriate practices towards the use of medications in either one of these two groups (SGLT-2i and GLP-1RA). Participants received two points for ‘Always,’ one point for ‘Sometimes,’ and zero for the ‘Never’ response.

Two items were intentionally included in the practice section to capture contextual information beyond the composite score: one assessed physician drug preference between SGLT-2i and GLP-1RA when clinical benefits and risks are similar, and the other assessed perceived barriers related to medication availability and patient accessibility. These items were excluded from the composite score because they reflect contextual factors rather than discrete physician behaviors amenable to a correct/incorrect scoring framework; both were retained and reported descriptively. A minimum score of 13 points out of 16 (>80%) was considered good to indicate safe and appropriate practice. Cut-off thresholds for classifying knowledge, attitude, and practice scores are conventional and vary across studies, commonly ranging from 60% to 80% of the maximum score (23–25). We therefore selected a threshold *a priori* for each construct: knowledge was classified as adequate and attitude as positive at more than 60% of the maximum score, whereas practice was classified as appropriate only at more than 80%. The stricter threshold for practice reflects that prescribing behavior is the clinical endpoint with direct patient-safety implications; in this rapidly evolving therapeutic area, with new agents and frequently updated guidelines, safe and guideline-concordant prescribing warrants a higher standard than declarative knowledge or attitudinal endorsement. Certain items needed to be reverse scored to ensure consistency in the overall analysis. For the ‘knowledge’ section, items 2, 5, 7, and 8 for SGLT-2i and items 11, 13, and 16 for GLP-1RA were reverse-scored. In the ‘attitude’ section, items 2 and 4 were also reverse scored to reflect negative perspectives accurately.

### Data collection

The data were collected by multiple means to ensure the reach to the largest number of the targeted population. Physicians from the targeted specialties were invited for participation through text messages or the chatting application “WhatsApp^®^.” We also communicated with the Saudi Commission for Health Specialties and requested their assistance by sending the electronic survey to physicians from these specialties based on their registries for physicians in Saudi Arabia. The message sent had a link to the questionnaire, which could be completed on the cell phones of the participants. Data was collected over a 12-month period, from December 2024 to November 2025, using an online survey tool (Microsoft Forms). The purpose of the questionnaire, voluntary participation and the anonymity of the respondents were clearly stated on the first page of the online questionnaire. Because the survey was disseminated through open WhatsApp broadcast messages and through the Saudi Commission for Health Specialties mass electronic distribution to registered physicians, the total number of physicians who received the invitation could not be determined; consequently, a precise response rate could not be calculated.

### Statistical analysis

Descriptive statistics were used to summarize participant characteristics and responses to the KAP items. Categorical variables were reported as frequencies and percentages, and continuous composite scores as means with standard deviations (SD). Composite scores were calculated for knowledge, with two sub-scores: 8 items for SGLT-2i and 8 items for GLP-1RA, scored 1 for correct and 0 for incorrect or “I am not sure”; for attitude, 9 items, each rated on a 5-point Likert scale from -2 ‘Totally Disagree’ to +2 ‘Totally Agree,’ with items 2 and 4 reverse-scored; and for practice, 8 items, scored 0 = never, 1 = sometimes, 2 = always. Two practice items were excluded from the composite: Q9 assessed a subjective drug preference without an evidence-based correct answer, and Q10 assessed a perceived environmental barrier rather than a physician behavior; both were retained and reported descriptively.

Internal consistency was quantified using the Kuder-Richardson formula 20 (KR-20) for the binary knowledge scales and Cronbach’s α for the ordinal attitude and practice scales. Spearman rank correlation coefficients (*ρ*) were computed to examine associations between the knowledge, attitude, and practice composite scores; the Spearman method was selected because the composite scores were discrete with a small number of unique values and did not satisfy the normality assumption required by Pearson correlation (Shapiro-Wilk *p* < 0.001 for all four scores). Bivariate associations between demographic characteristics and each dichotomized KAP outcome were examined using Pearson’s χ² test, with Fisher’s exact test substituted when expected cell counts were <5; the Bonferroni correction was applied to account for multiple comparisons.

To identify factors independently associated with each KAP outcome, multivariable logistic regression was performed. Because this was a cross-sectional study in which knowledge, attitude, and practice were measured concurrently, no causal ordering was assumed between these constructs; instead, a symmetric modelling approach was used in which each dichotomized KAP outcome (inadequate knowledge of SGLT-2i, inadequate knowledge of GLP-1RA, positive attitude, and inappropriate practice) was regressed on the demographic variables (age group, gender, region, and professional status) together with the continuous composite scores of the other two KAP constructs. Specialty was excluded from the multivariable models because of complete collinearity with professional status (all general practitioners had no sub-specialty). For each predictor, univariate logistic regression was first performed separately against each outcome to obtain unadjusted (crude) odds ratios; adjusted odds ratios were then obtained from the multivariable models. All estimates are reported with 95% confidence intervals (CI).

Because two outcomes had a low number of events (inadequate knowledge of SGLT-2i, n= 36 events; inappropriate practice, n= 98 events), Firth’s penalised-likelihood logistic regression was used for those models to reduce small-sample bias and prevent separation. Multicollinearity among predictors was assessed using the generalized variance inflation factor (GVIF), with GVIF^(1/(2·Df)) < 2.5 considered acceptable. All statistical tests were two-sided and a p-value <0.05 was considered statistically significant. Analyses were performed in R (version 4.3.1; R Foundation for Statistical Computing, Vienna, Austria) using the ‘psych,’ ‘logistf,’ ‘car,’ ‘broom,’ and ‘openxlsx’ packages.

## Results

### Participant characteristics

A total of 399 physicians completed the questionnaire. The largest age group was 25–35 years (150/399; 37.6%), followed by 46–55 years (122/399; 30.6%), and no participants fell in the over-65 category. Male physicians comprised 54.1% (216/399) and most respondents practiced in the Riyadh region (286/399; 71.7%). By professional status, specialists were the largest group (238/399; 59.6%), followed by residents (99/399; 24.8%). Among those reporting a sub-specialty, family medicine (177) and internal medicine (114) were the most common sub-specialties. Full demographic characteristics are presented in **Table 1**.

**Table 1:** Demographic characteristics for the participants (n = 399).

Characteristic	Frequency (%)
Age group, years	
25-35	150 (37.6)
36-45	75 (18.8)
46-55	122 (30.6)
56-65	52 (13.0)
Gender	
Male	216 (54.1)
Female	183 (45.9)
Region	
Riyadh	286 (71.7)
Other regions	113 (28.3)
Professional status	
General practitioner	48 (12.0)
Resident	99 (24.8)
Specialist	238 (59.6)
Consultant	14 (3.5)
Specialty (of those with a sub-specialty, n = 351)	
Family medicine	177 (50.4)
Internal medicine	114 (32.5)
Endocrinology	37 (10.5)
Cardiology	15 (4.3)
Nephrology	8 (2.3)

Numbers are presented as frequency (percentage).

### Reliability of the KAP scales

Internal consistency was acceptable for most scales: KR-20 was 0.61 for the 8-item SGLT-2i knowledge scale and 0.37 for the 8-item GLP-1RA knowledge scale. Cronbach’s α was 0.70 for the 9-item attitude scale and 0.80 for the 8-item practice scale (**Supplementary Table 1**).

### KAP composite scores and dichotomized outcomes

Mean composite scores (± SD) were 6.78 ± 1.41 (range 2-8) for knowledge about SGLT-2i, 5.54 ± 1.33 (range 1-8) for knowledge about GLP-1RA, 7.62 ± 3.60 (range -10 to 18) for attitude, and 13.94 ± 2.28 (range 7-16) for practice (**Supplementary Table 2**). Applying the pre-specified thresholds, adequate knowledge of SGLT-2i was demonstrated by 363 of 399 participants (91.0%), adequate knowledge of GLP-1RA by 313 (78.4%), positive attitude by 94 (23.6%), and appropriate practice by 301 (75.4%) (**Table 2**).

**Table 2:** Knowledge, attitude and practices (KSP) outcomes, with additional practice items distributions (n=399).

Characteristic	Frequency (%)
KAP outcomes (dichotomized)	
Adequate knowledge of SGLT-2i (≥5/8)	363 (91.0)
Adequate knowledge of GLP-1RA (≥5/8)	313 (78.4)
Positive overall attitude (≥11/18)	94 (23.6)
Appropriate practice (≥13/16)	301 (75.4)
Additional practice items (reported separately)*	
Drug preference for SGLT-2i over GLP-1RA when benefits and risks are similar (Q9)	
Always	142 (35.6)
Sometimes	235 (58.9)
Never	22 (5.5)
Perceived medication availability and patient accessibility as prescribing barriers (Q10)	
Always	359 (90.0)
Sometimes	34 (8.5)
Never	6 (1.5)

Numbers are presented as frequency (percentage).

^*^ Q9 and Q10 were excluded from the Practice composite score. Q9 assessed a subjective drug preference without an evidence-based correct answer, and Q10 assessed a perceived environmental barrier rather than a physician’s behavior; both are reported descriptively.

Regarding drug preference, 142 participants (35.6%) reported always preferring SGLT-2i over GLP-1RA when benefits and risks are similar, 235 (58.9%) sometimes, and 22 (5.5%) never. Regarding access barriers, 359 participants (90.0%) reported that medication availability and patient accessibility are always a common barrier to prescribing SGLT-2i or GLP-1RA (**Table 2**).

### Correlations between KAP constructs

All four composite scores were positively and significantly correlated (all p < 0.001). The strongest association was between attitude and practice (*ρ* = 0.67), followed by the correlation between the two knowledge sub-scores (*ρ* = 0.64). SGLT-2i knowledge correlated with attitude (*ρ* = 0.59) and practice (*ρ* = 0.57), and GLP-1RA knowledge correlated with attitude (*ρ* = 0.49) and practice (*ρ* = 0.43) (**Supplementary Table 3**).

### Bivariate associations between demographics and KAP outcomes

**Figure 1:**
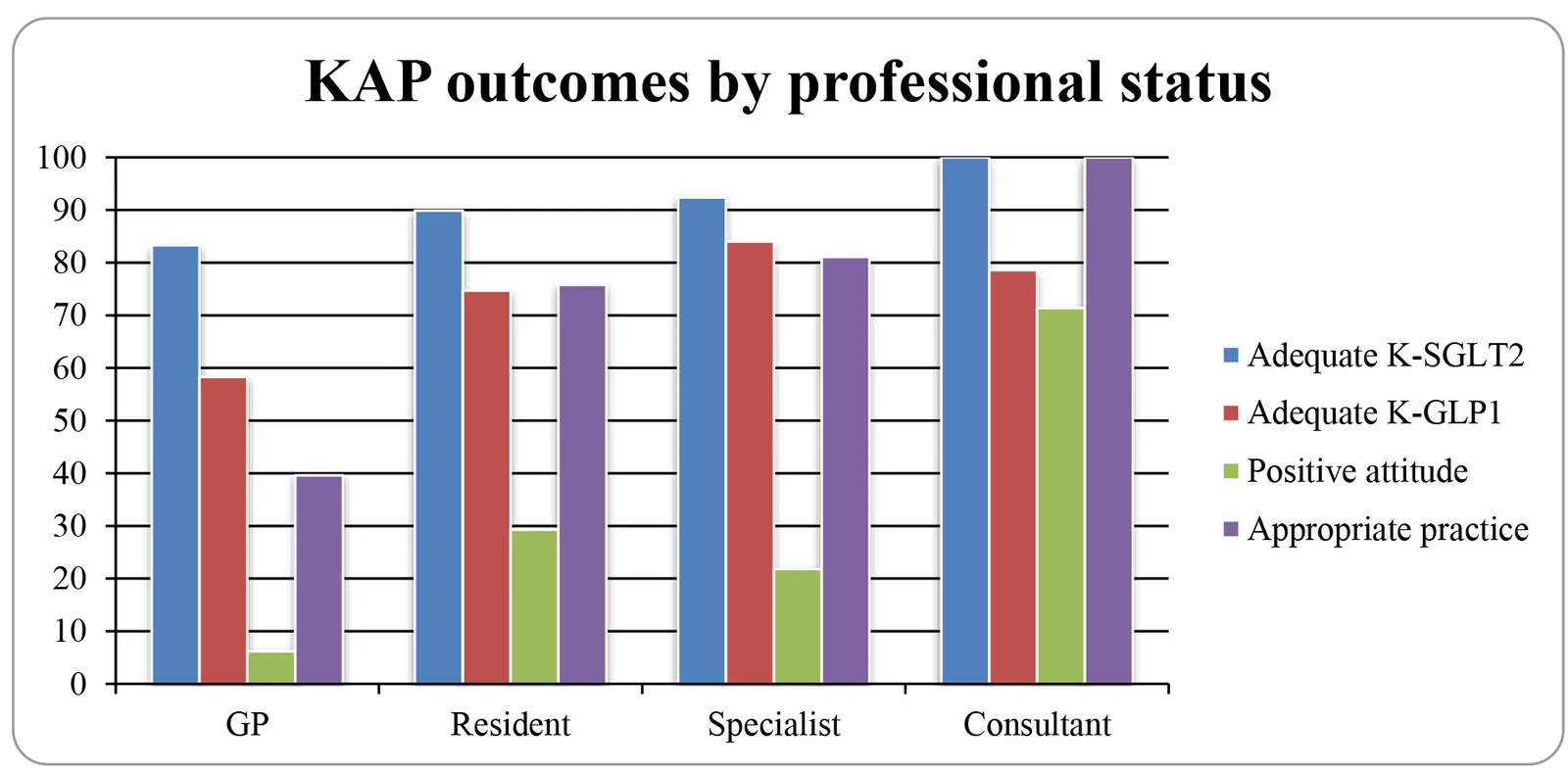
Proportion of physicians meeting each KAP threshold stratified by professional status (n= 399). Bars represent the percentage of physicians in each professional category (general practitioner, resident, specialist, and consultant) meeting the pre-specified threshold for adequate SGLT-2i knowledge, adequate GLP-1RA knowledge, positive attitude, and appropriate practice. SGLT-2i, sodium-glucose cotransporter-2 inhibitors; GLP-1RA, glucagon-like peptide-1 receptor agonist; KAP, knowledge, attitudes, and practices.

After Bonferroni correction for multiple comparisons, several demographic characteristics remained significantly associated with the KAP outcomes. Age group was associated with appropriate practice (*p* = 0.008). Region was associated with positive attitude (*p* < 0.001) and appropriate practice (*p* = 0.012). Professional status was associated with adequate knowledge of GLP-1RA (*p* = 0.026), positive attitude (*p* = 0.002), and appropriate practice (*p* = 0.002). Specialty was associated with positive attitude (*p* = 0.002) and appropriate practice (*p* = 0.002). Gender was not significantly associated with any KAP outcome. The proportions of physicians meeting each KAP threshold by professional status are shown in **Figure 1**; detailed bivariate results are presented in **Supplementary Tables 4-8**.

### Independent factors associated with KAP outcomes from multivariable analysis

**Table 3** presents the adjusted odds ratios from the four multivariable logistic regression models. All models had acceptable multicollinearity (GVIF^(1/(2·Df)) < 1.34). Visual summaries of the adjusted odds ratios from each model are provided in **Supplementary Figures 6-9**.

**Table 3:** Factors associated with KAP outcomes: adjusted odds ratios from four multivariable logistic regression models (n = 399).

Predictor (reference)	Inadequate K-SGLT2^a^ aOR (95% CI)	Inadequate K-GLP1RA aOR (95% CI)	Positive attitude aOR (95% CI)	Inappropriate practice^a^ aOR (95% CI)
Age group (ref: 25–35 years)				
36-45	0.81 (0.21-3.03)	1.77 (0.73-4.39)	1.62 (0.68-3.87)	**4.13 (1.11-16.5)**
46-55	0.46 (0.13-1.62)	**0.30 (0.12-0.77)**	1.38 (0.53-3.61)	**3.63 (1.09-13.7)**
56-65	**0.15 (0.02-0.77)**	**0.18 (0.05-0.58)**	2.28 (0.72-7.18)	**8.68 (2.34-37.2)**
Gender (ref: Male)				
Female	1.26 (0.58-2.78)	0.80 (0.45-1.39)	0.87 (0.50-1.52)	0.59 (0.31-1.09)
Region (ref: Riyadh)				
Other	1.65 (0.59-4.53)	1.06 (0.53-2.11)	**2.11 (1.07-4.16)**	**0.37 (0.13-0.99)**
Professional status (ref: GP)				
Resident	0.93 (0.24-3.79)	0.51 (0.18-1.39)	3.41 (0.78-15.0)	1.05 (0.29-3.92)
Specialist	1.86 (0.60-6.60)	0.58 (0.26-1.32)	1.83 (0.47-7.17)	**0.23 (0.09-0.57)**
Consultant	1.22 (0.01-18.1)	0.78 (0.13-4.02)	**14.6 (2.27-93.9)**	0.09 (0.00-1.17)
Knowledge SGLT-2i (per 1-point)	—	—	1.10 (0.82-1.49)	**0.70 (0.52-0.93)**
Knowledge GLP-1RA (per 1-point)	—	—	1.27 (0.96-1.68)	0.92 (0.68-1.27)
Attitude (per 1-point)	**0.75 (0.62-0.88)**	**0.79 (0.70-0.88)**	—	**0.68 (0.58-0.78)**
Practice (per 1-point)	**0.74 (0.61-0.90)**	**0.82 (0.70-0.95)**	**1.96 (1.49-2.59)**	—

aOR, adjusted odds ratio; CI, confidence interval; GP, general practitioner; K-SGLT2, knowledge of SGLT-2i; K-GLP1RA, knowledge of GLP-1RA. ^a^Firth’s penalized-likelihood logistic regression was used for the inadequate SGLT-2i knowledge model (n= 36 events) and the inappropriate practice model (n= 98 events) to reduce small-sample bias and prevent separation; standard logistic regression was used for the positive attitude model (n= 94 events). Reference categories are shown in parentheses; Bold values indicate significance; dashes (—) indicate that the variable is the outcome of the corresponding model. Each model adjusted for all other variables listed in the table.

### Factors associated with inadequate knowledge of SGLT-2i

In the adjusted Firth regression (**Table 3**; **Supplementary Table 9**), three factors were independently associated with inadequate SGLT-2i knowledge: older age, which was inversely associated (for 56–65 vs. 25–35 years, aOR= 0.15, 95%CI 0.02-0.77; *p* = 0.022), higher attitude score (aOR= 0.75 per one-point increase; *p* < 0.001), and higher practice score (aOR = 0.74 per one-point increase; *p* = 0.002).

### Factors associated with inadequate knowledge of GLP-1RA

Older age was inversely associated with inadequate GLP-1RA knowledge (for 46–55 vs. 25–35 aOR= 0.30, *p* = 0.013; for 56–65 vs. 25–35 aOR= 0.18, *p* = 0.005). Each one-point increase in attitude score (aOR= 0.79, *p* < 0.001) and in practice score (aOR= 0.82, *p* = 0.010) was associated with reduced odds of inadequate GLP-1RA knowledge (**Table 3**; **Supplementary Table 10**).

### Factors associated with positive attitude

Three factors were independently associated with greater odds of positive attitude toward SGLT-2i and GLP-1RA: practicing outside Riyadh (aOR= 2.11, 95%CI 1.07-4.16; *p* = 0.032), being a consultant compared with a general practitioner (aOR= 14.60, 95% CI 2.27-93.93; *p* = 0.005), and each one-point increase in practice score (aOR= 1.96, 95%CI 1.49-2.59; *p* < 0.001). Neither knowledge score retained independent significance after adjustment, although GLP-1RA knowledge showed a borderline association (aOR= 1.27, *p* = 0.096), suggesting that bivariate associations with attitude were largely explained by shared variance with practice (**Table 3**; **Supplementary Table 11**).

### Factors associated with inappropriate practice

Older age was associated with higher odds of inappropriate practice after adjustment, with the strongest effect observed in the oldest age group (**Table 3**; **Supplementary Table 12**). Relative to physicians aged 25–35 years, the adjusted odds of inappropriate practice were 4.13-fold higher in those aged 36–45 years (*p* = 0.034), 3.63-fold higher in those aged 46–55 years (*p* = 0.035), and 8.68-fold higher in those aged 56–65 years (95%CI 2.34-37.21; *p* < 0.001). Practicing outside Riyadh was associated with lower odds of inappropriate practice (aOR= 0.37; *p* = 0.047), as was being a specialist compared with a general practitioner (aOR= 0.23, 95%CI 0.09-0.57; *p* = 0.001). A higher SGLT-2i knowledge score (aOR= 0.70 per one-point increase; *p* = 0.013) and attitude score (aOR= 0.68 per one-point increase; *p* < 0.001) were both independently associated with lower odds of inappropriate practice. GLP-1RA knowledge was not independently associated with practice in the adjusted model (aOR= 0.92; *p* = 0.627).

## Discussion

This nationwide cross-sectional survey of 399 Saudi physicians yielded three principal findings. Theoretical knowledge of these two medication classes was generally high, with 91% of respondents demonstrating adequate knowledge of SGLT-2i and 78.4% of GLP-1RA. Despite this knowledge base, only 23.6% of physicians held an overall positive attitude toward these medications, even though 75.4% met the threshold for appropriate prescribing practice. Finally, the four KAP constructs were strongly intercorrelated, and several demographic and professional factors, namely older age, region, professional status, and the composite KAP scores, emerged as independent determinants of these outcomes after multivariable adjustment.

The high level of SGLT-2i knowledge observed here contrasts with the more modest estimates reported in earlier Saudi work. Somaili et al., surveying physicians in the southern region of Saudi Arabia, found that only a minority of respondents demonstrated adequate familiarity with the cardio-renal indications and contraindications of SGLT-2i (21). The improvement evident in our sample likely reflects repeated updates to the American Diabetes Association and European Association for the Study of Diabetes guidelines (2, 22), accumulating long-term outcome data from CVOTs (3–6), and intensified educational activity at the regional level. National continuing medical education initiatives, hospital-based grand rounds, industry-sponsored academic activities, and Ministry of Health awareness campaigns have all expanded the exposure of Saudi physicians to evidence on these agents. The convergence of these channels offers a plausible explanation for the higher knowledge baseline. Knowledge of GLP-1RA was nonetheless consistently lower than that of SGLT-2i. A comparable gap was reported by Shubrook et al. in a survey of United States primary care physicians, in which awareness of cardiovascular benefits lagged behind awareness of glycemic effects, particularly for newer injectable agents (18). The relatively recent expansion of the evidence base for GLP-1RA, including more recent cardiovascular and renal outcome trials (3, 4), may explain why familiarity has not yet caught up with that of the longer-established SGLT-2i class.

The most clinically meaningful finding in this study is the dissociation between adequate knowledge and overall positive attitude. Although nine of every ten physicians answered the majority of SGLT-2i knowledge items correctly, fewer than one in four held an overall positive attitude toward these medications. Exposure to guideline content and trial evidence does not, in itself, generate the professional conviction required to incorporate these agents into routine prescribing, and the finding argues against framing physician hesitancy as fundamentally a knowledge problem. This pattern is not unique to Saudi Arabia. Kanumilli et al., in a global survey of more than 1,677 physicians, reported a parallel disconnect in which clinicians were aware of cardio-renal evidence yet remained reluctant to initiate therapy, citing access restrictions, missed appointments, and concerns about adverse effects (19). Gao et al. similarly described physician-reported barriers including limited comfort and experience prescribing these drug classes, reimbursement and prior-authorization challenges, and the perception that initiating these medications belongs to a different specialty (17). In a more recent single-system survey, Yaseen and Lahiri found that over 80% of providers cited high cost and prior authorization requirements as the principal barriers to prescribing SGLT-2i and GLP-1RA, with limited prescribing experience also reported among cardiology and nephrology respondents (26). In our cohort, the near-universal endorsement of medication availability and patient accessibility as a prescribing barrier, with 90.0% of physicians reporting that this was always a constraint, points to contextual factors as important contributors to attitudinal hesitancy, alongside knowledge.

Despite this widespread attitudinal hesitancy, three quarters of respondents met the threshold for appropriate practice. This figure is markedly higher than the 19% utilization reported by Korayem et al. in their two-center retrospective review of eligible patients in Riyadh (15), and substantially higher than the 7.2% prescription rate reported by McCoy et al. in a United States cohort (8). These comparisons should be drawn cautiously, since the practice composite reflects self-reported behavior rather than dispensing data, and self-report tends to overestimate guideline-concordant prescribing. The pattern of physicians reporting frequent appropriate behaviors while expressing reservations about the medications themselves is consistent with the concept of guideline-concordant but reluctant prescribing previously described in qualitative work from Hong Kong (14) and in the United Kingdom CPRD analysis by Farmer et al. (12). Mahtta et al., analyzing patients with established atherosclerotic cardiovascular disease and type 2 diabetes in the Veterans Affairs system, demonstrated marked facility-level variation in SGLT-2i and GLP-1RA utilization that could not be explained by patient-level factors alone (27). Taken together, these findings reinforce a multilevel interpretation in which institutional context shapes prescribing as strongly as individual physician characteristics.

To contextualize the access barriers reported by participants, it is useful to summarize the prescribing environment in Saudi Arabia. Both SGLT-2i and GLP-1RA, including oral semaglutide, are registered with the Saudi Food and Drug Authority and are commercially available (28, 29). In the public sector, comprising Ministry of Health hospitals, primary care centers, and other governmental facilities, these agents are provided to eligible patients at no direct cost but are subject to institutional formulary listing and, for some agents, protocol-based or specialty-linked initiation criteria; affordability and availability have improved following the introduction of generic SGLT-2i (16). In the private sector, access depends on out-of-pocket payment or private insurance coverage, and the higher acquisition cost of these agents, particularly GLP-1RA, can constrain uptake. Prescribing is not restricted by regulation to any single specialty, and any licensed physician managing T2DM may prescribe these medications; in practice, however, initiation is more concentrated in specialist settings. These structural features help explain why 90.0% of respondents identified medication availability and patient accessibility as a constant prescribing barrier.

A noteworthy descriptive finding was the preference for SGLT-2i over GLP-1RA when benefits and risks were perceived as similar, with 35.6% of physicians always preferring SGLT-2i and only 5.5% never doing so. The oral route of administration, lower acquisition cost, broader cardiovascular and renal indication base (5, 6), and greater clinical familiarity may all contribute to this preference. Patient-level factors are also relevant, since injection avoidance is a recognized determinant of treatment acceptance in the region and physicians may anticipate this when selecting between classes. This consideration has become more nuanced with the availability of oral semaglutide, which is registered with the Saudi Food and Drug Authority and is increasingly used in local practice (29, 30). Because our questionnaire assessed GLP-1RA as a single class, it could not capture whether the availability of an oral formulation modified physician attitudes or preferences, and the growing use of oral GLP-1RA may have attenuated injection-related reservations for some respondents.

The multivariable analyses revealed an apparent paradox in the relationship between physician age and the KAP outcomes. Physicians aged 56 to 65 years showed substantially lower odds of inadequate knowledge of both SGLT-2i and GLP-1RA, yet exhibited an almost ninefold increase in the adjusted odds of inappropriate practice compared with their youngest colleagues. A similar though weaker pattern was observed in the 36-to-45 and 46-to-55 year groups. This finding is consistent with the well-described phenomenon of clinical or therapeutic inertia, in which accumulated experience and entrenched cognitive routines lead established clinicians to adhere to familiar regimens even when their declarative knowledge of newer therapies is sound (19, 20). In a landmark retrospective cohort study of more than 80,000 patients with type 2 diabetes, Khunti et al. showed that many patients remained in poor glycemic control for years before treatment intensification, with the delay largely independent of patient characteristics and pointing to prescriber-side determinants (31). The implication is that interventions limited to knowledge transfer are unlikely to be sufficient for more senior physicians, and that strategies addressing decision support, audit-and-feedback, and clinical workflow redesign will be needed to translate knowledge into prescribing change. A separate clinical concern relates to treatment persistence. Physicians who prescribe under institutional pressure rather than personal conviction may be less likely to maintain therapy when minor or transient adverse effects arise. This raises questions about the long-term realization of the cardio-renal benefits demonstrated in the CVOTs (3–6).

Geographic location also emerged as an independent factor, with physicians practicing outside Riyadh showing approximately twofold higher odds of a positive attitude and significantly lower odds of inappropriate practice. Although this might appear counterintuitive given the concentration of tertiary academic centers in the capital, patient-mix and workload differences offer a plausible explanation. Physicians outside major referral centers often manage less complex T2DM populations, which may allow more sustained attention to individual prescribing decisions, whereas the concentration of complex multi-comorbid patients in Riyadh may amplify perceived risk and reduce willingness to initiate newer agents. Differences in formulary access and the balance of primary care and specialty practice across regions may further contribute. These interpretations remain speculative and merit dedicated mixed-methods investigation.

The association between professional status and KAP outcomes echoes findings from international surveys (17, 18, 26). Specialists and consultants in our cohort were considerably more likely to demonstrate appropriate practice than general practitioners, and consultants in particular showed a marked increase in the odds of a positive attitude (aOR=14.60, 95%CI 2.27-93.93). The wide confidence interval reflects the small consultant subgroup (n= 14) and should temper any quantitative interpretation. The qualitative pattern, in which seniority and specialty training are linked to more favorable engagement with SGLT-2i and GLP-1RA, is consistent with the greater clinical exposure of specialists to high-risk patients with established cardiovascular or renal disease, who constitute much of the primary evidence base for these agents. Lower attitude and practice scores among general practitioners should not be read as a deficit in competence; primary care physicians manage a broader and less severe patient mix and may apply a more conservative threshold for therapy intensification. From a workforce planning standpoint, however, primary care providers are the first point of contact for most patients with T2DM in Saudi Arabia and therefore represent the most productive focus for educational and decision-support interventions. Disparities in access to newer antidiabetic agents have also been documented at the patient level. Eberly et al. showed lower SGLT-2i use among commercially insured Black patients and those with lower household incomes in the United States, despite consistent guideline recommendations (32). This observation underscores that organizational and structural factors shape who receives these therapies, a dynamic equally relevant to the Saudi context.

The strong intercorrelation among the KAP constructs, with the highest coefficient observed between attitude and practice (*ρ* = 0.67), supports the theoretical premise of the KAP framework that beliefs and behavior are tightly coupled. In the multivariable models, both knowledge of SGLT-2i and overall attitude remained independent predictors of appropriate practice, whereas knowledge of GLP-1RA did not retain significance after adjustment for shared variance with the other constructs. This pattern suggests that translating knowledge into practice is mediated, at least in part, by physician confidence and conviction. Interventions aimed solely at improving factual knowledge of GLP-1RA, without addressing attitudinal barriers, are therefore unlikely to alter prescribing behavior. Targeted small-group case-based learning, patient-narrative-based education, and academic detailing focused on real-world cardiovascular and renal benefits are likely to prove more effective than passive didactic formats.

Within Saudi Arabia, earlier KAP work has produced a notably different picture. Aldhobaib et al., surveying healthcare professionals at Qassim University, reported good knowledge (73.5%), good attitude (78.5%), and good practice (80.3%) regarding GLP-1RA (33). Although the knowledge and practice estimates from that study broadly align with ours, the contrast in attitude scores is striking; several explanations are plausible. The Qassim study included a smaller, single-institution sample that combined physicians, pharmacists, and nurses, whereas our cohort was restricted to practicing physicians and drew from multiple regions and practice settings. Differences in instrument structure, scoring conventions, and dichotomization thresholds may also contribute. More importantly, our attitudinal items focused not only on familiarity but also on prescribing confidence, perceived risk, and willingness to discuss benefits and risks with patients. These dimensions may be more sensitive to the contextual barriers that have intensified over the intervening years, including drug pricing and supply constraints. The discrepancy supports the view that physician attitudes toward these medications are not stable over time and may be highly responsive to shifts in the prescribing environment.

Several implications follow from these findings. Educationally, structured continuing medical education programs targeting primary care physicians and more senior clinicians, with an emphasis on integrating SGLT-2i and GLP-1RA into routine T2DM management for patients with cardiovascular and renal indications, should be prioritized. Because our data indicate that prescribing patterns often reflect adherence to institutional protocols rather than individual clinical conviction, educational efforts should aim explicitly at building confidence and trust in these therapies, not at memorization of guideline criteria. At the policy level, the near-universal report of access barriers indicates that any educational intervention will yield limited returns unless paired with formulary reform, expanded reimbursement, and patient affordability initiatives, particularly given the demonstrated long-term cardio-renal benefits of these classes (3–6). Health system stakeholders, including the Saudi Ministry of Health and the Saudi Commission for Health Specialties, are well placed to lead combined educational and structural interventions and to integrate dispensing-level surveillance to monitor their impact, as has been demonstrated in larger administrative cohorts internationally (27, 32).

This study has several strengths. It is among the largest physician KAP surveys on SGLT-2i and GLP-1RA conducted in Saudi Arabia, with multi-region recruitment and broad coverage of specialties. The questionnaire was developed iteratively by a multidisciplinary team, subjected to face-validity review by independent practicing physicians, and pilot tested prior to deployment. The analytical strategy combined descriptive, bivariate, and multivariable approaches, with Firth’s penalized regression applied to outcomes with low event counts to mitigate small-sample bias and prevent separation, and with multicollinearity formally evaluated. The use of a symmetric multivariable framework, in which each KAP outcome was modeled as a function of the others, avoided arbitrary causal assumptions inappropriate to a cross-sectional design.

### Limitations

This study has several limitations. The internal consistency of the GLP-1RA knowledge subscale was modest (KR-20 = 0.37), suggesting that the eight items may have measured heterogeneous content domains, and the dichotomized classification of adequate GLP-1RA knowledge should therefore be interpreted with caution. Future iterations of the instrument should refine or expand this subscale and re-evaluate its psychometric properties. The cross-sectional design precludes causal inference, particularly for the observed associations between knowledge, attitude, and practice. Self-reported prescribing practice is subject to social desirability bias and may overestimate the proportion of physicians prescribing in line with guidelines; linkage to dispensing or claims data, including those available through hospital pharmacy systems and the Saudi Food and Drug Authority, would substantially strengthen inference in future work. Recruitment relied on electronic dissemination, including via the Saudi Commission for Health Specialties, which may have favored participation by physicians more interested in or comfortable with diabetes pharmacotherapy. In addition, the recruitment strategy did not permit calculation of a denominator of invited physicians, so a formal response rate could not be reported, and the potential for self-selection cannot be excluded. Although nationwide in scope, the sample was concentrated in Riyadh (71.7%), and findings may not generalize equally to all regions of the Kingdom. Specialty was excluded from the multivariable models because of collinearity with professional status, and the consultant subgroup was small (n= 14), limiting the precision of subgroup-specific estimates. The questionnaire evaluated GLP-1RA as a single class and did not distinguish injectable from oral formulations; given the availability of oral semaglutide in Saudi Arabia, future instruments should assess knowledge, attitudes, and practices separately for injectable and oral GLP-1RA. Finally, the pre-specified KAP cut-off thresholds, although consistent with prior literature, remain conventional rather than empirically derived, and sensitivity analyses using alternative thresholds would add robustness in future work.

## Conclusion

In this nationwide survey, Saudi physicians demonstrated generally adequate theoretical knowledge of SGLT-2i and, to a lesser extent, GLP-1RA, and approximately three quarters reported appropriate prescribing practice. However, only a minority held an overall positive attitude toward these agents, and access-related barriers were near-universally endorsed. The most consistent independent correlates of inappropriate practice were older age, practicing in Riyadh, general practitioner status, and lower SGLT-2i knowledge and attitude scores, while higher attitude and practice scores were the most consistent correlates of adequate knowledge. These cross-sectional findings are consistent with under-prescribing being associated more with attitudinal hesitancy and structural access barriers than with gaps in physician knowledge; however, the study design precludes causal inference. Future efforts should combine targeted continuing professional development for primary care and more senior physicians with policy-level action on medication availability and affordability. Longitudinal studies linking physician KAP profiles to objective dispensing data, together with multicenter qualitative research exploring why guideline-aware physicians remain attitudinally hesitant, will be needed to close the gap between evidence, perception, and practice in cardiometabolic care for patients with T2DM.

## Data Availability

The raw data supporting the conclusions of this article will be made available by the authors, without undue reservation.
