# Supplementary material for: Knowledge, attitudes, and practices of Saudi physicians toward the use of SGLT-2 inhibitors and GLP-1 receptor agonists in patients with type 2 diabetes mellitus: a nationwide cross-sectional study
Source: Front Endocrinol (Lausanne). 2026 Aug 27;17:1929125. doi: 10.3389/fendo.2026.1929125 (PMC13559532; doi:10.3389/fendo.2026.1929125)
Supplement: Supplementary file 1 [file SupplementaryFile1.docx]

| Table S1. Internal consistency of KAP scales | | |
| --- | --- | --- |
| Scale | **Reliability Index** | **Value** |
| Knowledge SGLT-2i (8 items) | KR-20 | 0.61 |
| Knowledge GLP-1RA (8 items) | KR-20 | 0.37 |
| Attitude (9 items, -2 to +2 scoring) | Cronbach's α | 0.70 |
| Practice (8 items; drug preference and access barrier items excluded) | Cronbach's α | 0.80 |

| **Table S2.** Descriptive statistics of composite scores | | | | | | | |
| --- | --- | --- | --- | --- | --- | --- | --- |
| **Score** | **Mean ± SD** | **Median** | **Min** | **Max** | **Theoretical max** | **Cutoff for positive** | **N positive (%)** |
| Knowledge SGLT-2i | 6.78 ±1.41 | 7 | 2 | 8 | 8 | ≥5 (>60%) | 363 (91.0) |
| Knowledge GLP-1RA | 5.54 ±1.33 | 6 | 1 | 8 | 8 | ≥5 (>60%) | 313 (78.4) |
| Attitude | 7.62 ±3.60 | 7 | -10 | 18 | 18 | ≥11 (>60%) | 94 (23.6) |
| Practice | 13.94 ±2.28 | 15 | 7 | 16 | 16 | ≥13 (>80%) | 301 (75.4) |

| **Table S3.** Spearman rank correlation coefficients (ρ) with p-values (two-sided) | | | | |
| --- | --- | --- | --- | --- |
| **Variable** | **K-SGLT2** | **K-GLP1** | **Attitude** | **Practice** |
| K-SGLT2 | 1 | 0.636  **<0.001** | 0.585  **<0.001** | 0.569  **<0.001** |
| K-GLP1 | 0.636  **<0.001** | 1 | 0.489  **<0.001** | 0.432  **<0.001** |
| Attitude | 0.585  **<0.001** | 0.489  **<0.001** | 1 | 0.672  **<0.001** |
| Practice | 0.569  **<0.001** | 0.432  **<0.001** | 0.672  **<0.001** | 1 |

| **Table S4.** Bivariate associations (Bonferroni-adjusted) | | | | | |
| --- | --- | --- | --- | --- | --- |
| **Predictor** | **Outcome** | **Test** | **Statistic** | ***p*-value (raw)** | ***p*-value**  **(Bonferroni-adjusted)** |
| Age group | Adequate K-SGLT-2i | Fisher (MC) |  | 0.5478 | 1 |
| Age group | Adequate K-GLP-1RA | Chi-square | 11.05 | **0.0115** | 0.230 |
| Age group | Positive attitude | Chi-square | 10.06 | **0.0181** | 0.362 |
| Age group | Appropriate practice | Chi-square | 18.42 | **0.0004** | **0.008** |
| Gender | Adequate K-SGLT-2i | Chi-square | 0 | 0.9968 | 1 |
| Gender | Adequate K-GLP-1RA | Chi-square | 0.93 | 0.3353 | 1 |
| Gender | Positive attitude | Chi-square | 0.73 | 0.3924 | 1 |
| Gender | Appropriate practice | Chi-square | 1.08 | 0.2993 | 1 |
| Region | Adequate K-SGLT-2i | Chi-square | 0 | 1 | 1 |
| Region | Adequate K-GLP-1RA | Chi-square | 0 | 1 | 1 |
| Region | Positive attitude | Chi-square | 29.88 | **<0.001** | **<0.001** |
| Region | Appropriate practice | Chi-square | 11.71 | **0.0006** | **0.012** |
| Professional status | Adequate K-SGLT-2i | Fisher (MC) |  | 0.1643 | 1 |
| Professional status | Adequate K-GLP-1RA | Fisher (MC) |  | **0.0013** | **0.026** |
| Professional status | Positive attitude | Fisher (MC) |  | **0.0001** | **0.002** |
| Professional status | Appropriate practice | Fisher (MC) |  | **0.0001** | **0.002** |
| Specialty | Adequate K-SGLT-2i | Fisher (MC) |  | **0.0103** | 0.206 |
| Specialty | Adequate K-GLP-1RA | Fisher (MC) |  | **0.0268** | 0.536 |
| Specialty | Positive attitude | Fisher (MC) |  | **0.0001** | **0.002** |
| Specialty | Appropriate practice | Fisher (MC) |  | **0.0001** | **0.002** |

| **Table S5.** Univariate: Inadequate K-SGLT2 vs demographics (Firth) | | |
| --- | --- | --- |
| **Predictor^*^** | **Crude OR (95% CI)** | **Crude p-value** |
| Age 36-45 | 0.89 (0.34-2.15) | 0.8056 |
| Age 46-55 | 0.84 (0.37-1.85) | 0.6672 |
| Age 56-65 | 0.40 (0.08-1.36) | 0.1537 |
| Female | 0.94 (0.47-1.86) | 0.8686 |
| Other regions | 1.00 (0.45-2.06) | 0.9943 |
| Resident | 0.56 (0.21-1.52) | 0.2488 |
| Specialist | 0.40 (0.17-1.01) | 0.0513 |
| Consultant | 0.16 (0.00-1.47) | 0.1226 |
| ^*^Reference categories: Age 25-35 years, Male, Riyadh, General practitioner." |  |  |

| **Table S6.** Univariate: Inadequate K-GLP-1RA vs demographics | | |
| --- | --- | --- |
| **Predictor^*^** | **Crude OR (95% CI)** | **Crude p-value** |
| Age 36-45 | 1.44 (0.77-2.64) | 0.2446 |
| Age 46-55 | **0.53 (0.28-0.97)** | **0.0449** |
| Age 56-65 | 0.48 (0.18-1.09) | 0.0969 |
| Female | 0.77 (0.47-1.24) | 0.2783 |
| Other regions | 0.97 (0.56-1.64) | 0.9234 |
| Resident | **0.47 (0.23-0.98)** | **0.0448** |
| Specialist | **0.27 (0.14-0.52)** | **<0.001** |
| Consultant | 0.38 (0.08-1.41) | 0.1776 |
| ^*^Reference categories: Age 25-35 years, Male, Riyadh, General practitioner." |  |  |

| **Table S7.** Univariate: Positive Attitude vs demographics | | |
| --- | --- | --- |
| **Predictor^*^** | **Crude OR (95% CI)** | **Crude p-value** |
| Age 36-45 | 1.21 (0.66-2.22) | 0.5347 |
| Age 46-55 | **0.47 (0.25-0.86)** | **0.0158** |
| Age 56-65 | 0.54 (0.23-1.16) | 0.1300 |
| Female | 0.79 (0.50-1.27) | 0.3307 |
| Other regions | **3.85 (2.38-6.25)** | **<0.001** |
| Resident | **6.25 (2.04-25.00)** | **0.0041** |
| Specialist | **4.17 (1.45-16.67)** | **0.0201** |
| Consultant | **33.33 (8.33->100)** | **<0.001** |
| ^*^Reference categories: Age 25-35 years, Male, Riyadh, General practitioner." |  |  |

| **Table S8.** Univariate: Inappropriate Practice vs demographics (Firth) | | |
| --- | --- | --- |
| **Predictor^*^** | **Crude OR (95% CI)** | **Crude p-value** |
| Age 36-45 | 0.93 (0.44-1.87) | 0.8384 |
| Age 46-55 | 1.61 (0.91-2.86) | 0.1007 |
| Age 56-65 | **3.69 (1.88-7.31)** | **0.0002** |
| Female | 0.76 (0.48-1.21) | 0.2510 |
| Other regions | **0.35 (0.18-0.62)** | **0.0002** |
| Resident | **0.21 (0.10-0.44)** | **<0.001** |
| Specialist | **0.16 (0.08-0.30)** | **<0.001** |
| Consultant | **0.02 (0.00-0.19)** | **<0.001** |
| ^*^Reference categories: Age 25-35 years, Male, Riyadh, General practitioner." | | |

| **Table S9.** Full crude and adjusted odds ratios for inadequate knowledge of SGLT-2i (Firth's penalised regression; n=399, 36 events) | | | | |
| --- | --- | --- | --- | --- |
| **Predictor** | **Crude OR (95% CI)** | **Crude p** | **Adjusted OR (95% CI)** | **Adjusted p** |
| Age 36-45 | 0.89 (0.34-2.15) | 0.806 | 0.81 (0.21-3.03) | 0.760 |
| Age 46-55 | 0.84 (0.37-1.85) | 0.667 | 0.46 (0.13-1.62) | 0.220 |
| Age 56-65 | 0.40 (0.08-1.36) | 0.154 | **0.15 (0.02-0.77)** | **0.022** |
| Female | 0.94 (0.47-1.86) | 0.869 | 1.26 (0.58-2.78) | 0.556 |
| Other regions | 1.00 (0.45-2.06) | 0.994 | 1.65 (0.59-4.53) | 0.332 |
| Resident | 0.56 (0.21-1.52) | 0.249 | 0.93 (0.24-3.79) | 0.913 |
| Specialist | 0.40 (0.17-1.01) | 0.051 | 1.86 (0.60-6.60) | 0.293 |
| Consultant | 0.16 (0.00-1.47) | 0.123 | 1.22 (0.01-18.08) | 0.907 |
| Attitude (per 1-point) | **0.68 (0.58-0.77)** | **<0.001** | **0.75 (0.62-0.88)** | **<0.001** |
| Practice (per 1-point) | **0.65 (0.56-0.75)** | **<0.001** | **0.74 (0.61-0.90)** | **0.002** |
| *OR = odds ratio; CI = confidence interval. Reference categories: Age 25-35 years, Male, Riyadh, General practitioner. Crude estimates are from univariate models (one predictor at a time); adjusted estimates are from the symmetric multivariable Firth regression including all listed variables simultaneously.* | | | | |

| **Table S10.** Full crude and adjusted odds ratios for inadequate knowledge of GLP-1RA (logistic regression; n= 399, 86 events) | | | | |
| --- | --- | --- | --- | --- |
| **Predictor** | **Crude OR (95% CI)** | **Crude p** | **Adjusted OR (95% CI)** | **Adjusted p** |
| Age 36-45 | 1.44 (0.77-2.64) | 0.245 | 1.77 (0.73-4.39) | 0.210 |
| Age 46-55 | **0.53 (0.28-0.97)** | **0.045** | **0.30 (0.12-0.77)** | **0.013** |
| Age 56-65 | 0.48 (0.18-1.09) | 0.097 | **0.18 (0.05-0.58)** | **0.005** |
| Female | 0.77 (0.47-1.24) | 0.278 | 0.80 (0.45-1.39) | 0.424 |
| Other regions | 0.97 (0.56-1.64) | 0.923 | 1.06 (0.53-2.11) | 0.876 |
| Resident | **0.47 (0.23-0.98)** | **0.045** | 0.51 (0.18-1.39) | 0.187 |
| Specialist | **0.27 (0.14-0.52)** | **<0.001** | 0.58 (0.26-1.32) | 0.188 |
| Consultant | 0.38 (0.08-1.41) | 0.178 | 0.78 (0.13-4.02) | 0.774 |
| Attitude (per 1-point) | **0.76 (0.69-0.82)** | **<0.001** | **0.79 (0.70-0.88)** | **<0.001** |
| Practice (per 1-point) | **0.73 (0.66-0.81)** | **<0.001** | **0.82 (0.70-0.95)** | **0.010** |
| *OR = odds ratio; CI = confidence interval. Reference categories: Age 25-35 years, Male, Riyadh, General practitioner. Crude estimates are from univariate models (one predictor at a time); adjusted estimates are from the symmetric multivariable regression including all listed variables simultaneously.* | | | | |

| **Table S11.** Full crude and adjusted odds ratios for positive attitude (logistic regression; n= 399, 94 events) | | | | |
| --- | --- | --- | --- | --- |
| **Predictor** | **Crude OR (95% CI)** | **Crude p** | **Adjusted OR (95% CI)** | **Adjusted p** |
| Age 36-45 | 1.21 (0.66-2.22) | 0.535 | 1.62 (0.68-3.87) | 0.276 |
| Age 46-55 | **0.47 (0.25-0.86)** | **0.016** | 1.38 (0.53-3.61) | 0.510 |
| Age 56-65 | 0.54 (0.23-1.16) | 0.130 | 2.28 (0.72-7.18) | 0.161 |
| Female | 0.79 (0.50-1.27) | 0.331 | 0.87 (0.50-1.52) | 0.621 |
| Other regions | **3.85 (2.38-6.25)** | **<0.001** | **2.11 (1.07-4.16)** | **0.032** |
| Resident | **6.25 (2.04-25.00)** | **0.004** | 3.41 (0.78-15.03) | 0.104 |
| Specialist | **4.17 (1.45-16.67)** | **0.020** | 1.83 (0.47-7.17) | 0.383 |
| Consultant | **33.33 (8.33->100)** | **<0.001** | **14.60 (2.27-93.93)** | **0.005** |
| Knowledge SGLT-2i (per 1-point) | **1.92 (1.52-2.50)** | **<0.001** | 1.10 (0.82-1.49) | 0.523 |
| Knowledge GLP-1RA (per 1-point) | **1.79 (1.45-2.27)** | **<0.001** | 1.27 (0.96-1.68) | 0.096 |
| Practice (per 1-point) | **2.17 (1.75-2.86)** | **<0.001** | **1.96 (1.49-2.59)** | **<0.001** |
| *OR = odds ratio; CI = confidence interval. Reference categories: Age 25-35 years, Male, Riyadh, General practitioner. Crude estimates are from univariate models (one predictor at a time); adjusted estimates are from the symmetric multivariable regression including all listed variables simultaneously.* | | | | |

| **Table S12s.** Full crude and adjusted odds ratios for inappropriate practice (Firth's penalised regression; N = 399, 98 events) | | | | |
| --- | --- | --- | --- | --- |
| **Predictor** | **Crude OR (95% CI)** | **Crude p** | **Adjusted OR (95% CI)** | **Adjusted p** |
| Age 36-45 | 0.93 (0.44-1.87) | 0.838 | **4.13 (1.11-16.54)** | **0.034** |
| Age 46-55 | 1.61 (0.91-2.86) | 0.101 | **3.63 (1.09-13.71)** | **0.035** |
| Age 56-65 | **3.69 (1.88-7.31)** | **<0.001** | **8.68 (2.34-37.21)** | **<0.001** |
| Female | 0.76 (0.48-1.21) | 0.251 | 0.59 (0.31-1.09) | 0.094 |
| Other regions | **0.35 (0.18-0.62)** | **<0.001** | 0.37 (0.13-0.99) | 0.047 |
| Resident | **0.21 (0.10-0.44)** | **<0.001** | 1.05 (0.29-3.92) | 0.941 |
| Specialist | **0.16 (0.08-0.30)** | **<0.001** | **0.23 (0.09-0.57)** | **0.001** |
| Consultant | **0.02 (0.00-0.19)** | **<0.001** | 0.09 (0.00-1.17) | 0.068 |
| Knowledge SGLT-2i (per 1-point) | **0.46 (0.38-0.55)** | **<0.001** | **0.70 (0.52-0.93)** | **0.013** |
| Knowledge GLP-1RA (per 1-point) | **0.51 (0.42-0.62)** | **<0.001** | 0.92 (0.68-1.27) | 0.627 |
| Practice (per 1-point) | **0.57 (0.49-0.64)** | **<0.001** | **0.68 (0.58-0.78)** | **<0.001** |
| *OR = odds ratio; CI = confidence interval. Reference categories: Age 25-35 years, Male, Riyadh, General practitioner. Crude estimates are from univariate models (one predictor at a time); adjusted estimates are from the symmetric multivariable regression including all listed variables simultaneously.* | | | | |

**Figure S1.** Proportion of physicians answering each SGLT-2i knowledge item correctly (n= 399).

Each bar represents the percentage of physicians who correctly answered one of the eight SGLT-2i knowledge items (items 1-8). Items 2, 5, 7, and 8 were negatively worded and were reverse scored prior to analysis. SGLT-2i: sodium-glucose cotransporter-2 inhibitors.

**Figure S2.** Proportion of physicians answering each GLP-1RA knowledge item correctly (n= 399).

Each bar represents the percentage of physicians who correctly answered one of the eight GLP-1RA knowledge items (items 9-16). Items 11, 13, and 16 were negatively worded and were reverse scored prior to analysis. GLP-1RA: glucagon-like peptide-1 receptor agonists.

Knowledge: SGLT-2i (% correct)

|  | % correct |
| --- | --- |
| K-SGLT2 #1 | 98 |
| K-SGLT2 #2 | 83.5 |
| K-SGLT2 #3 | 95.5 |
| K-SGLT2 #4 | 65.4 |
| K-SGLT2 #5 | 86.5 |
| K-SGLT2 #6 | 92.7 |
| K-SGLT2 #7 | 65.2 |
| K-SGLT2 #8 | 91.7 |

Knowledge: GLP-1RA (% correct)

|  | % correct |
| --- | --- |
| K-GLP1 #9 | 94.5 |
| K-GLP1 #10 | 91.5 |
| K-GLP1 #11 | 63.2 |
| K-GLP1 #12 | 84.5 |
| K-GLP1 #13 | 49.1 |
| K-GLP1 #14 | 82.7 |
| K-GLP1 #15 | 13 |
| K-GLP1 #16 | 75.4 |

**Figure S4.** Proportion of physicians responding "Always" to each practice item (n= 399).

Each bar represents the percentage of physicians who responded "Always" to one of the eight practice items included in the composite score. Items PP #9 and PP #10 were excluded from the composite and are not shown here (see Table 2). SGLT-2i: sodium-glucose cotransporter-2 inhibitors; GLP-1RA: glucagon-like peptide-1 receptor agonists.

**Figure S3.** Proportion of physicians responding positively to each attitude item (n= 399).

Each bar represents the percentage of physicians who scored above zero (i.e., responded "Agree" or "Totally Agree") on each of the nine attitude items. Items 2 and 4 were negatively worded and were reverse-scored so that a positive score consistently reflects a favorable attitude toward SGLT-2i and GLP-1RA. SGLT-2i: sodium-glucose cotransporter-2 inhibitors; GLP-1RA: glucagon-like peptide-1 receptor agonists.

Practice (% 'Always' responses)

|  | % Always |
| --- | --- |
| PP #1 | 86.5 |
| PP #2 | 70.7 |
| PP #3 | 63.9 |
| PP #4 | 89 |
| PP #5 | 78.7 |
| PP #6 | 48.9 |
| PP #7 | 89.7 |
| PP #8 | 79.4 |

Attitude (% positive responses)

|  | % positive |
| --- | --- |
| Attitude #1 | 90.5 |
| Attitude #2 | 7.3 |
| Attitude #3 | 92.5 |
| Attitude #4 | 54.9 |
| Attitude #5 | 96.2 |
| Attitude #6 | 91.5 |
| Attitude #7 | 67.7 |
| Attitude #8 | 84 |
| Attitude #9 | 64.7 |

**Figure S6.** Adjusted odds ratios for factors associated with inadequate knowledge of SGLT-2i (Firth's penalised logistic regression; n= 399; 36 events).

Bars represent the adjusted odds ratio (aOR) for each predictor. The dashed line at OR = 1 indicates no association. Reference categories: Age 25-35 years, Male, Riyadh, General practitioner. Full numeric results with 95% confidence intervals and *p*-values are provided in Supplementary Table S9. SGLT-2i: sodium-glucose cotransporter-2 inhibitors.

**Figure S5**. Proportion of physicians meeting each KAP threshold, stratified by age group (n= 399).

Bars represent the percentage of physicians in each age group (25-35, 36-45, 46-55, and 56-65 years) meeting the pre-specified threshold for adequate SGLT-2i knowledge (≥5/8), adequate GLP-1RA knowledge (≥5/8), positive attitude (≥11/18), and appropriate practice (≥13/16). SGLT-2i: sodium-glucose cotransporter-2 inhibitors; GLP-1RA: glucagon-like peptide-1 receptor agonists; KAP: knowledge, attitudes, and practices.

Inadequate K-SGLT2 - adjusted OR

|  |  |
| --- | --- |
| Age 36-45 | 0.81 |
| Age 46-55 | 0.46 |
| Age 56-65 | 0.15 |
| Female | 1.26 |
| Other regions | 1.65 |
| Resident | 0.93 |
| Specialist | 1.86 |
| Consultant | 1.22 |
| Attitude (per 1-pt) | 0.75 |
| Practice (per 1-pt) | 0.74 |

KAP outcomes by age group

|  | Adequate K-SGLT2 | Adequate K-GLP1 | Positive attitude | Appropriate practice |
| --- | --- | --- | --- | --- |
| 25-35 | 89.3 | 75.3 | 28 | 81.3 |
| 36-45 | 90.7 | 68 | 32 | 82.7 |
| 46-55 | 91 | 85.2 | 15.6 | 73 |
| 56-65 | 96.2 | 86.5 | 17.3 | 53.8 |

**Figure S8.** Adjusted odds ratios for factors associated with positive attitude toward SGLT-2i and GLP-1RA (logistic regression; n= 399; 94 events).

Bars represent the adjusted odds ratio (aOR) for each predictor. The dashed line at OR = 1 indicates no association. Reference categories: Age 25-35 years, Male, Riyadh, General practitioner. Full numeric results with 95% confidence intervals and p-values are provided in Supplementary Table S11. SGLT-2i: sodium-glucose cotransporter-2 inhibitors; GLP-1RA: glucagon-like peptide-1 receptor agonists.

**Figure S7.** Adjusted odds ratios for factors associated with inadequate knowledge of GLP-1RA (logistic regression; n= 399; 86 events).

Bars represent the adjusted odds ratio (aOR) for each predictor. The dashed line at OR = 1 indicates no association. Reference categories: Age 25-35 years, Male, Riyadh, General practitioner. Full numeric results with 95% confidence intervals and *p*-values are provided in Supplementary Table S10. GLP-1RA: glucagon-like peptide-1 receptor agonists.

Psitive attitude - adjusted OR

|  |  |
| --- | --- |
| Age 36-45 | 0.62 |
| Age 46-55 | 0.72 |
| Age 56-65 | 0.44 |
| Female | 1.15 |
| Other regions | 0.47 |
| Resident | 0.29 |
| Specialist | 0.55 |
| Consultant | 0.07 |
| K-SGLT2 (per 1-pt) | 0.91 |
| K-GLP1 (per 1-pt) | 0.79 |
| Practice (per 1-pt) | 0.51 |

Inadequate K-GLP1 - adjusted OR

|  |  |
| --- | --- |
| Age 36-45 | 1.77 |
| Age 46-55 | 0.3 |
| Age 56-65 | 0.18 |
| Female | 0.8 |
| Other regions | 1.06 |
| Resident | 0.51 |
| Specialist | 0.58 |
| Consultant | 0.78 |
| Attitude (per 1-pt) | 0.79 |
| Practice (per 1-pt) | 0.82 |

**Figure S9.** Adjusted odds ratios for factors associated with inappropriate prescribing practice (Firth's penalised logistic regression; N = 399; 98 events).

Bars represent the adjusted odds ratio (aOR) for each predictor. The dashed line at OR = 1 indicates no association. Reference categories: Age 25-35 years, Male, Riyadh, General practitioner. Full numeric results with 95% confidence intervals and *p*-values are provided in Supplementary Table S12. SGLT-2i: sodium-glucose cotransporter-2 inhibitors; GLP-1RA: glucagon-like peptide-1 receptor agonists.

Inappropriate practice - adjusted OR

|  |  |
| --- | --- |
| Age 36-45 | 4.13 |
| Age 46-55 | 3.63 |
| Age 56-65 | 8.68 |
| Female | 0.59 |
| Other regions | 0.37 |
| Resident | 1.05 |
| Specialist | 0.23 |
| Consultant | 0.09 |
| K-SGLT2 (per 1-pt) | 0.7 |
| K-GLP1 (per 1-pt) | 0.92 |
| Attitude (per 1-pt) | 0.68 |
